# Impact of educational intervention on knowledge regarding ocular exercises for computer vision syndrome

**DOI:** 10.6026/973206300210583

**Published:** 2025-04-30

**Authors:** Mahalakshmi B., Siva Subramanian N., Beula S.S, Chaudhari Unnatiben Pravinbhai, Darji Dixitkumar Kiritbhai, Riddhiben Jashvantji, Chaudhari Ravina Babulal, Chaudhari Ridhdhiben Govinbhai, Dave Jimi Vikasbhai, Chaudhari Priyankaben Bharatbhai

**Affiliations:** 1 Department of Paediatric Nursing, Nootan College of Nursing, Sankalchand Patel University, Visnagar, Gujarat - 384315, India; 2 Department of Psychiatric Nursing, Nootan College of Nursing, Sankalchand Patel University, Visnagar, Gujarat - 384315, India; 3 Department of Community Health Nursing, Nootan College of Nursing, Sankalchand Patel University, Visnagar, Gujarat - 384315 India

**Keywords:** Video-assisted teaching program, computer vision syndrome, ocular exercises, adolescents, digital eye strain

## Abstract

The increasing use of digital devices has led to a rise in computer vision syndrome (CVS), especially among adolescents. Prolonged screen exposure results in symptoms like eye strain, dryness, irritation and headaches, affecting students' health and academic performance. Therefore, it is of interest to evaluate the effectiveness of video-assisted teaching programs in enhancing knowledge about ocular exercises for computer vision syndrome prevention. A pre-experimental, one-group pre-test post-test research design was employed, involving 60 adolescents aged 13-18 years from selected schools in Visnagar. Participants were assessed using a structured knowledge questionnaire before and after exposure to video-assisted teaching programs. Data showed a significant increase in knowledge post-intervention (p < 0.001). The percentage of participants with adequate knowledge increased from 0% to 86.66%, while those with inadequate knowledge decreased from 70% to 0%.

## Background:

With the increasing reliance on digital devices such as computers, tablets and smartphones, there has been a significant rise in cases of computer vision syndrome (CVS), a condition characterized by eye strain, dryness, irritation and other visual disturbances due to prolonged screen exposure [1]. Adolescents, in particular, are at heightened risk due to excessive screen time related to academic activities and entertainment [2]. Computer vision syndrome has been reported to negatively affect not only ocular health but also academic performance and overall well-being [3]. Several studies have highlighted the importance of preventive strategies, including proper screen ergonomics, frequent breaks and ocular exercises to alleviate symptoms of computer vision syndrome [4]. Ocular exercises have been demonstrated to enhance visual comfort and reduce the incidence of computer vision syndrome related symptoms by improving the strength and endurance of the ocular muscles [5]. However, the lack of awareness and knowledge about these exercises remains a significant challenge among adolescents [6]. Video-assisted teaching programs (VATP) have emerged as an effective intervention in improving knowledge and behavioural changes regarding health-related issues, including ocular care [7]. These educational interventions utilize audiovisual elements to engage learners, thereby enhancing retention and practical application of knowledge. Studies suggest that VATP can significantly improve awareness and adherence to ocular exercise routines, leading to reduced computer vision syndrome prevalence among students [8]. Therefore, it is of interest to assess the effectiveness of a video-assisted teaching program on knowledge regarding ocular exercises for computer vision syndrome among adolescents in selected schools in Visnagar.

## Methodology:

A quantitative, pre-experimental research design was adopted to evaluate the effectiveness of the Video-Assisted Teaching Program (VATP) on ocular exercises among adolescents. The study used a one-group pre-test and post-test design to measure the impact of the intervention.

[1] Population and sampling: The study included 60 adolescents aged 13-18 years from selected schools in Visnagar, chosen through a convenience sampling technique.

[2] Data collection: A structured questionnaire was used to assess participants' knowledge before and after the intervention. The questionnaire included demographic details and knowledge-based multiple-choice questions about ocular exercises and CVS.

[3] Intervention: Participants underwent a VATP session that provided audiovisual content on ocular exercises, prevention of computer vision syndrome and best practices for screen use.

[4] Data analysis: Descriptive statistics were used to summarize the demographic data and knowledge scores. A paired t-test was conducted to compare the pre-test and post-test scores, determining the significance of knowledge improvement.

[5] Ethical considerations: Approval was obtained from the relevant institutional review board and informed consent was taken from participants and their guardians before participation.

## Results:

Table 1 show that majority of participants (58.33%) were aged 16-18 years, with more males (58.33%) than females. Most students were in 9th grade (33.33%) and resided in urban areas (50%). Smartphones (50%) were the most commonly used devices, indicating high digital exposure among adolescents. Table 2 shows the association between post-test knowledge scores and demographic variables. Age (p=0.05) and education level (p=0.04) had a significant impact on knowledge improvement, indicating that older students and those in higher grades benefited more from the video-assisted teaching program. However, gender, residence and device usage did not show a statistically significant association, suggesting that knowledge improvement was not influenced by these factors. Figure 1 - (see PDF) shows the comparison of knowledge scores before and after the video-assisted teaching program. The percentage of participants with inadequate knowledge significantly dropped from 70% to 0%, while those with adequate knowledge increased from 0% to 86.66%, indicating a marked improvement in knowledge levels post-intervention.

## Discussion:

The findings of this study demonstrate the effectiveness of Video-Assisted Teaching Programs (VATP) in improving adolescents' knowledge regarding ocular exercises for Computer Vision Syndrome (CVS). The significant increase in post-test knowledge scores (p < 0.001) suggests that audiovisual learning methods are a powerful tool for enhancing awareness and retention of health-related knowledge. The substantial increase in adequate knowledge (0% to 86.66%) and the complete elimination of inadequate knowledge (70% to 0%) highlight the success of VATP in this context. Our study shows that the proportion of students with adequate knowledge increased from 0% to 86.66% after the VATP intervention, suggesting its strong impact. This aligns with the findings of Shende *et al.* where video-assisted teaching programs significantly improved knowledge scores about ocular exercises in school children [9]. That means that one more study conducted by Kumar *et al.* found that medical coding trainees showed significant knowledge improvement (p < 0.001) after video teaching interventions on computer vision syndrome [10]. This supports the present study's conclusion that structured audiovisual educational programs can enhance knowledge and awareness of computer vision syndrome prevention. That is supported by Habeeb *et al.*, who found that structured teaching interventions significantly improved nursing students' awareness and understanding of CVS, reinforcing the effectiveness of targeted educational approaches [11]. Zhuang and Sitepu demonstrated that eye exercises significantly reduced symptoms of computer vision syndrome among medical students, further emphasizing the role of proactive ocular health education [12]. Furthermore, Halim *et al.* highlighted that ergonomic and environmental factors significantly influence the prevalence of CVS, suggesting that educational programs should also incorporate preventive strategies beyond exercises [13]. In support of the present study, James and Anand (2023) found that a Video-Assisted Teaching Program (VATP) on yoga eye exercises effectively improved knowledge and awareness among high school students, significantly reducing digital eye strain [14]. The mean difference between pre-test and post-test scores was statistically significant, confirming that structured VATP interventions can be a key tool in eye strain prevention education.

Similarly, Jayadev and Patel (2023) assessed the effectiveness of structured teaching programs on knowledge regarding computer vision syndrome among office employees in Mehsana district. Their study found that post-test knowledge scores were significantly higher than pre-test scores (p < 0.05), confirming the effectiveness of structured education in workplace settings [15]. This further supports the effectiveness of targeted teaching programs in reducing digital eye strain across different populations. Likewise, Prabu and Anbumani (2022) conducted a pre-experimental study on computer vision syndrome awareness among college students and found a significant improvement in post-test knowledge scores, similar to the current study's findings [16]. The increase in awareness highlights the need for incorporating computer vision syndrome education into routine college health programs. A study by George *et al.* (2021) evaluating computer vision syndrome knowledge among engineering students revealed that 70% of students had only average knowledge, underscoring the need for structured educational interventions [17]. This supports the argument that VATP can effectively bridge knowledge gaps and improve preventive measures among high-risk student groups. Finally, Sindhuja *et al.* (2016) studied the effectiveness of nursing interventions on computer vision syndrome knowledge among IT professionals and found that structured educational programs significantly reduced asthenopia (eye strain) and improved computer vision syndrome knowledge levels [18]. These findings highlight the broad applicability of structured teaching programs in preventing digital eye strain across different demographic groups.

## Strengths and limitations:

This study's major strength lies in its pre-test and post-test design, allowing for a clear assessment of VATP's effectiveness. Additionally, the study provides practical recommendations for integrating video-assisted learning into adolescent health education. However, a limitation is the lack of a control group, which could have provided stronger comparative evidence. Future research should employ randomized controlled trials (RCTs) to validate these findings. Another potential limitation is that long-term retention of knowledge was not assessed, highlighting the need for follow-up studies to evaluate sustained impact.

## Future research directions:

The results confirm that VATP is an effective tool for improving awareness about computer vision syndrome prevention, reinforcing the study's hypothesis. The significance of this study lays in its applicability to broader adolescent health education programs, making it an important step toward reducing digital eye strain in young populations. However, unanswered questions remain regarding long-term behavioral adherence to ocular exercises and the effectiveness of integrating VATP with other educational formats, such as interactive applications or gamified learning. Future research should focus on multi-modal educational strategies and explore their comparative effectiveness.

## Figures and Tables

**Table 1:** Demographic characteristics of participants

**Demographic Variable**	**Frequency (n=60)**	**Percentage (%)**
**Age (Years)**		
13-15	25	41.66%
16-18	35	58.33%
**Gender**		
Male	35	58.33%
Female	25	41.67%
**Education Level**		
9th Grade	20	33.33%
10th Grade	15	25.00%
11th Grade	13	21.66%
12th Grade	12	20.00%
**Residence**		
Urban	30	50.00%
Semi-Urban	20	33.33%
Rural	10	16.67%
Device Used		
Computer	20	33.33%
Smartphone	30	50.00%
Tablet	10	16.67%

**Table 2:** Association between post-test knowledge score and demographic variables

**Demographic Variable**	**Chi-Square Value**	**p-Value**	**Significance**
Age	1.64	0.05	Significant
Gender	0.065	0.07	Not Significant
Education Level	1.24	0.04	Significant
Residence	0.14	0.08	Not Significant
Device Used	0.57	0.06	Not Significant
